# The Th17/Treg Cell Balance: A Gut Microbiota-Modulated Story

**DOI:** 10.3390/microorganisms7120583

**Published:** 2019-11-20

**Authors:** Hongyu Cheng, Xiong Guan, Dekun Chen, Wentao Ma

**Affiliations:** Veterinary Immunology Laboratory, College of Veterinary Medicine, Northwest Agriculture and Forestry University, Yangling 712100, China; chenghongyu@nwsuaf.edu.cn (H.C.); 2016011033@nwafu.edu.cn (X.G.)

**Keywords:** gut microbiota, Th17, Treg, balance, metabolites

## Abstract

The intestinal tract of vertebrates is normally colonized with a remarkable number of commensal microorganisms that are collectively referred to as gut microbiota. Gut microbiota has been demonstrated to interact with immune cells and to modulate specific signaling pathways involving both innate and adaptive immune processes. Accumulated evidence suggests that the imbalance of Th17 and Treg cells is associated with the development of many diseases. Herein, we emphatically present recent findings to show how specific gut microbiota organisms and metabolites shape the balance of Th17 and Treg cells. We also discuss the therapeutic potential of fecal microbiota transplantation (FMT) in diseases caused by the imbalance of Th17 and Treg cells

## 1. Introduction

The vertebrate intestine harbors a complex and dynamic population of microorganisms, collectively known as the gut microbiota, which co-evolved with the host by maintaining a symbiotic relationship [1,2,3]. These microorganisms, including bacteria, fungi, and viruses, are present in numbers that are 10 times greater than the number of host cells [4,5]. Germ-free (GF) animals have provided clues to the importance of gut microbiota for health, as these animals exhibit various immune defects and higher susceptibility to infection [6,7]. Gut microbiota offer many benefits to the host through its regulation of host immunity [8,9]. For example, gut microbiota can regulate the differentiation and development of immune cells, as demonstrated by studies using GF mice and antibiotic treatment models, which have facilitated the discovery of many interesting phenomena [10]. Th17 and Treg cells are two vital lymphocyte subsets with opposing functions [11]. Abnormal ratio of Th17 and Treg have been frequently found to be a key feature of metabolic or immunologic disorder-associated diseases, including several types of chronic inflammations [12,13], allergic diseases [14,15], autoimmune diseases, and cancers [16,17]. A number of studies have shown that gut microbiota is closely associated with the balance of Th17 and Treg. Various research groups have reported that colonic Th17 cells and Tregs of GF mice are significantly decreased [18,19]. It has recently been found that gut microbiota-derived metabolites ATP and short-chain fatty acids (SCFAs) stimulate the differentiation and development of Th17 cells and Tregs, respectively [20,21,22]. Understanding the relationship between specific gut microbiota organisms and the balance of Th17 cells and Tregs would therefore benefit the cure of a range of diseases caused by the imbalance of these two cell subsets.

In recent years, the success of fecal microbiota transplantation (FMT) for the treatment of several diseases has made this therapy among the most discussed research area in the world. Until now, FMT has been proven an effective treatment for ulcerative colitis [23], Crohn’s disease [24], steroid-resistant acute graft-versus-host disease [25], hepatic encephalopathy [26], functional gastrointestinal disorders [27], and blood disorders [28]. However, it should be noted that, in many diseases, although the application of FMT is a promising treatment, many obstacles must still be overcome before this method can be fully used clinically. It is apparent that understanding the relationship between specific gut microbiota organisms and host health would benefit further development of FMT therapy.

Herein, we provide an overview of how specific components of gut microbiota and their metabolites shape the balance of Th17 cells and Tregs. We also highlight the relevant mechanisms underlying these observations and draw attention to clinical implications of gut microbiota modulation. In addition, we talked about the reported application of FMT therapy in several diseases caused by the imbalance of Th17 cells and Tregs.

## 2. Gut Microbiota

More than 100 trillion microorganisms inhabit skin and mucosal surfaces of animal hosts [29]. These microorganisms, including bacteria, fungi, and viruses, are present in numbers that are 10 times greater than the number of host cells [4,5] and co-evolve with their hosts by maintaining symbiotic relationships [30,31]. The effects of gut microbiota on human health have frequently been attributed to indirect and direct immunomodulatory activities [32,33]. In fact, disturbances of normal microbiome composition have been frequently found to be a key feature of metabolic or immunologic disorders [34,35], while accumulating evidence indicates that gut microbiota exhibit both pro-inflammatory and anti-inflammatory properties [36,37]. For example, different groups have reported that GF mice demonstrate decreased frequencies of Th17 cells and colonic Tregs [18,19]. More recently, researchers have found that gut microbiota-derived metabolites ATP and SCFAs stimulate the differentiation and development of Th17 and Tregs, respectively [20,21,22]. Indeed, host homeostasis is largely dependent upon gut microbiota, which functions through synergistic or antagonistic effects [29,38]. Thus, overall gut microbiota balance is vital and any change in its composition may disrupt the intestinal ecological balance, leading to the imbalance of the immune system. 

## 3. Specific Gut Microbiota Organisms Shapes the Balance of Th17 and Treg

Gut microbiota is required for the expansion and differentiation of intra-intestinal and systemic immune cells [39,40,41,42,43,44], with several recent studies showing that Th17 and Treg are decreased significantly in antibiotic-treated or germ-free animals, regardless of whether the animals were in a normal or diseased state [18,45,46,47]. Indeed, the balance of Th17 and Treg is abnormal in gut microbiota-depleted mice compared with mice with complete microbiota [39]. Although it is clear that both differentiation and functional maturation of immune cells depend largely on the presence of gut microbiota, the mechanisms by which the gut microbiome influences the balance of Th17 and Treg are still unknown.

### 3.1. Segmented Filamentous Bacteria

Intestinal lamina propria (LP) Th17 cells, which are indispensable for mucosal protection, are induced and maintained by *segmented filamentous bacteria* (SFB) [48,49,50,51,52]. Ivanov et al. examined the representation of SFB in Th17 cell-sufficient and Th17 cell-deficient mice and demonstrated that differentiation of Th17 cells correlates with the presence of SFB [53]. This result has been further supported by findings that the SFB attached to ileal epithelial cells could stimulate the production of reactive oxygen species (ROS), thus enhancing the secretion of IL-1β and promoting Th17 cell differentiation [54,55]. In addition, Ivanov et al. used real-time PCR and confirmed that all three serum amyloid A (SAA) isoforms were induced in the terminal ilea of GF mice upon colonization with SFB or SFB^+^ Jackson microbiota, but SAA were not induced only by Jackson microbiota alone [53]. Subsequently, further research found that SFB colonization induced the production of SAA proteins 1 and 2 (SAA1/2) in terminal ilea, and SAA acted on LP dendritic cells (DCs) to promote local IL-17A expression in RORγt^+^ T cell and Th17 cell differentiation in vitro [54,56]. In another line of research, CD11c**^+^**MHCII**^+^** monocyte-derived cells, a subset of antigen-presenting cells, have been shown to secrete considerable amounts of IL-1β when exposed to SFB in the intestine. Subsequently, the IL-1β functions together with IL-6, TGF-β and SAA to promote SFB-specific Th17 cell differentiation [48]. Meanwhile, Sano et al. observed increases in surface markers of DC maturation, including CD80, CD86, MHCII, and OX40L, upon exposure of bone marrow dendritic cells (BMDCs) to apo-SAA and showed that SAA may stimulate dendritic cells to produce IL-23 that sustains Th17 activation and survival [56]. Interestingly, Th17-derived IL-17 could in turn limit SFB expansion via the production of antimicrobial peptides (AMPs) and ROS [57,58]. Moreover, Flannigan et al. examined the entry of neutrophils into mouse ilea over the course of the first seven days of SFB colonization and demonstrated that colonization with SFB-containing microbiota induced durable recruitment of neutrophils into the ilea in an IL-17A- and CXCR2-dependent process [57]. We believe that this interesting phenomenon resembles the negative feedback regulation reported previously that serves to avoid damage to the body caused by immune hyperactivity and to maintain homeostasis of the host immune system.

### 3.2. Clostridia

*Clostridia* are one of the highest density of Gram-positive spore-forming gut microbiota in the small intestine [59]. *Clostridium* cluster IV and XIVa are most abundant in the cecum and proximal colon [19] and have been demonstrated to promote the induction of colonic Tregs and prevent inflammatory bowel disease, as identified using 16S rRNA sequences [60]. Previous studies have suggested that a specific fraction of intestinal Treg cells were differentiated under the effect of the gut microbiota [61]. Recently, Atarashi et al. isolated 17 Treg-cell-inducing gut microbiota strains that belonged to clusters IV, XIVa, and XVIII of *Clostridia* from the human indigenous microbiota [62]. Further oral administration of a combination of the 17 strains to adult mice resulted in attenuated disease severity of colitis and allergic diarrhea [14]. In addition, Treg cell accumulation in colonic LP can also be induced by *Clostridia* [19]. The mechanism for Treg enrichment may depend on the *Clostridium* cluster IV and XIVa production of SCFAs, which have multiple metabolic and immune functions [63]. Specifically, SCFAs can induce the production of TGF-β1 from epithelial cells and thus contribute to the de novo induction of peripheral Tregs [14]. Butyrate, one important member of SCFAs, can induce the proliferation of thymic Treg cells in a GPR4315 (belong to G protein-coupled receptor family)-dependent manner [64]. In addition, butyrate can inhibit histone deacetylase (HDAC) and promote the acetylation of histone H3 in the enhancer of Foxp3, resulting in the differentiation of naive CD4^+^ T cells into peripheral Treg cells [64]. In addition to its effects on Treg cells, butyrate can also suppress the activation of dendritic cells by inhibiting the expression of RelB of the NF-κB signaling pathway [65]. It has also been reported that butyrate can induce the expression of several anti-inflammatory genes in dendritic cells dependent on GPR109a [66]. Considering the key role of immunosuppression in many inflammatory diseases, further in-depth studies investigating the related mechanisms and exploring the interactions between *Clostridia* and other coexisting microbes, as well as their products are clearly needed.

### 3.3. Bacteroides fragilis

Toll-like receptors (TLRs) serve as pattern recognition receptors (PRRs) that recognize different but overlapping microbial components to eliminate pathogens [67]. Mounting evidence has shown that *Bacteroides fragilis* (*B. fragilis*), a species of gut microbiota, activates the TLR pathway of T lymphocytes to establish host-microbial symbiosis and influences T cell development and differentiation [68,69]. TLR2 deletion in CD4^+^ T cells results in anti-microbial immune responses that reduce *B. fragilis* colonization of its unique mucosal niche during homeostasis [70]. Specifically, TLR1, TLR2, and NOD2 are the main PRRs responsible for recognition of *B. fragilis* [71]. Notably, when stimulated with heat-killed *B. fragilis*, human peripheral blood mononuclear cells (PBMCs) produce high levels of IL-6 and IL-8, moderate levels of IL-1β and TNF-α, and low levels of IL-10, IL-17, IL-23 and IFN-γ [71]. 

The effect of *B. fragilis* is largely dependent on polysaccharide A (PSA), an immunomodulatory molecule present in the capsule of *B. fragilis* that participates in establishing host-microbial symbiosis and maintaining host immunity homeostasis [30,72,73]. Several studies have found that PSA is required for *B. fragilis* colonization and occupation of the mucosal niche through TLR2 signaling [68,74,75,76,77]. In line with these findings, Russler-Germain et al. demonstrated that TLR2 removal from the surface of CD4^+^ T cells resulted in anti-microbial immune responses that reduce *B. fragilis* colonization [78]. Conversely, intestinal dendritic cells can present PSA to CD4^+^ T cells, leading to appropriate cytokine production, which in turn enhances the ability of *B. fragilis* to influence host immune homeostasis [73]. Importantly, PSA is required for the adaptation of CD4^+^ T cells to become Foxp3^+^ Treg cells that produce IL-10, making it an effective preventive method and therapy for experimental colitis in mice [79]. It seems that the contribution of PSA to Treg lineage differentiation is dependent upon TLR2 present on the surfaces of CD4**^+^** T cells, as TLR2-deficient T cells failed to produce IL-10 when stimulated by PSA. These results therefore suggest that the TLR-mediated signaling pathway likely helps the establishment of commensal gut colonization. In line with this finding, several studies have shown that PSA suppressed differentiation of Th17 cells mainly through CD4**^+^** T cell-intrinsic TLR signaling [70,79,80,81]. In addition, PSA also inhibited Th17 cell differentiation via suppressing the production of Th17-inducing cytokines. For example, PSA on the surface of Bacteroides fragilis inhibited IL-1β-induced inflammation in human fetal enterocytes via TLR2 and TLR4 [82]. In another study, *Bacteroides fragilis*-induced macrophage IL-1β secretion was shown to upregulate the expression of hepcidin in the liver, a process dependent on the bone morphogenetic protein signaling pathway [83]. Interestingly, enterotoxigenic Bacteroides fragilis (ETBF), a specific strain of *B. fragilis* [84], exhibited several features that were not present in common *B. fragilis* staining, such as the production and secretion of colibactin (clbB) and Bacteroides fragilis toxin (bft) oncotoxins. Such activities appear to induce increased IL-17 production in the colon and DNA damage within the colonic epithelium that eventually promotes the development of early colon neoplasia [85].

### 3.4. Lactobacillus reuteri

**Figure 1:**
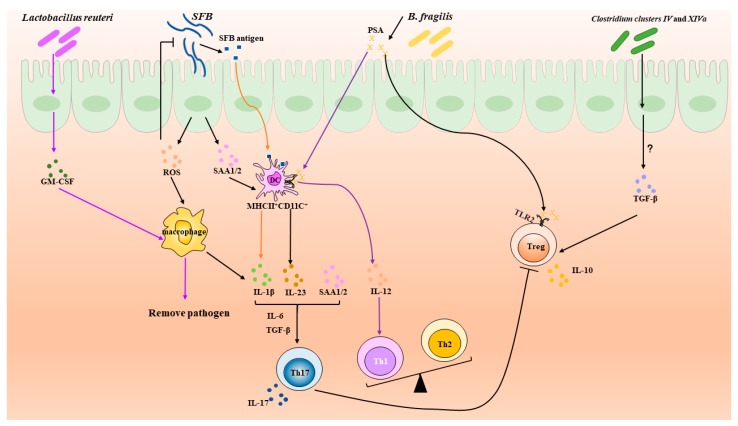
Roles of the microbiota in the balance of Th17 and Treg.

*Lactobacillus reuteri* (*L. reuteri*) is a Gram-positive bacterium that naturally inhabits in the mammalian gut [86]. Recently, *L. reuteri* strains have been used as probiotics to protect against systemic and respiratory infections caused by *Escherichia coli*, influenza virus, *Klebsiella pneumoniae*, *Listeria monocytogenes*, *Staphylococcus aureus* or *Streptococcus pneumoniae* [87,88,89,90]. Brown et al. found that gut granulocyte-macrophage colony-stimulating factor (GM-CSF) production in the lung in response to infections was dependent on commensal microbiota [91]. In addition, GM-CSF signaling is also required for the regulation of lung immunity in the upper airway via NOD-like receptor (NLR)-stimulating bacteria (Figure 1) [91]. Moreover, further research has demonstrated that *L. reuteri* can protect against lung infections induced by both Gram-negative and Gram-positive pathogens by stimulating GM-CSF production [91,92]. In addition to inhibiting lung infections, existing results indicate that *L. reuteri* also has a therapeutic effect on certain autoimmune diseases and metabolic disorders. In lupus-prone mice, Zhang et al. reported significantly decreased levels of *Lactobacillaceae* during lupus development. Interestingly, treatment with retinoic acid restored the level of *Lactobacilli* in these mice, a phenomenon accompanied by improved kidney functions in these mice [15]. Galley et al. showed that stress treatment reduced *Porphyromonadaceae* and *Lactobacillaceae* in the gut. Specifically, *L. reuteri* colonization was significantly decreased [93]. In addition to the above, *L. reuteri* is also associated with obesity [94], neurodevelopmental disorder [94] and so on. However, having a higher proportion of *L. reuteri* alone cannot guarantee greater bodily health, but having stable gut microbiota appears to be important for health. 

Gut microbiota is required for the expansion and differentiation of intra-intestinal and systemic immune cells. Reactive oxygen species (ROS) and serum amyloid A (SAA) produced by intestinal epithelial cells (IECs) in response to the adhesion of segmented filamentous bacteria (SFB) enhance secretion of IL-1β and IL-23 to promote Th17 cell development. SFB antigen plays a synergistic role in this process. Treg cells are induced by TGF-β, the production of which is stimulated by *Clostridia* clusters IV and XIVa, while the underlying mechanism is unclear. In addition, polysaccharide A (PSA) derived from B. fragilis promote Treg differentiation through TLR2 signaling. However, beyond that, PSA can also promote Th1 cell development to adjust balance of Th1 and Th2 via TLR2 signaling pathway on MHII^+^CD11C^+^ DC. *Lactobacillus reuteri*, a kind of probiotic, can stimulate the production of GM-CSF, which plays a key role in alveolar macrophage-mediated clearance of pathogens.

### 3.5. Bifidobacterium

*Bifidobacterium* is a genus belonging to the phylum *Actinobacteria*, which is one of the major phyla present in the intestinal tract of healthy humans [95]. *Bifidobacterium* strains are mainly divided into four different species, *Bifidobacterium longum*, *Bifidobacterium breve*, *Bifidobacterium bifidum,* and *Bifidobacterium animalis spp.* [96]. *Bifidobacterium* are a pioneering early colonizer of the gut [97] and play key roles in human immune system maturation, mainly through interacting directly with immune cells or through modulating specific pathways involved in innate and adaptive immune processes (Table 1) [98,99,100].

*Bifidobacterium* can induce DC maturation and species/strain-dependent T cell polarization [95,101,102]. In in-vitro models, all *Bifidobacterium longum* strains can trigger increased production of IL-10 and TNF-α, although strains of the same species also induced variable cytokine patterns. For example, the strain *B. longum W11* can strongly enhance the production of Th1 cytokines, while the strains within the same species i.e., *B. longum NCIMB 8809* and *BIF53*, induce low levels of Th1 cytokines [103]. Meanwhile, all *Bifidobacteria* have been shown to induce full DC maturation, but with differences in levels of cytokine production. *B. animalis* and *B. longum* induced secretion of large amounts of IFN-γ and TNF-α, in agreement with the Th1 cytokine profile observed after DC maturation; however, *B. bifidum* induced poor secretion of these cytokines with significant amounts of IL-17 [104], and after DC stimulation high levels of IL-1β/IL-12 ratio was observed [96]. Thus, *Bifidobacteria* are not only involved in inflammation, but are also associated with host immune regulation.

In in vivo models, *Bifidobacteria* species have also been demonstrated to play important roles in the balance between Th1 and Th2 responses, the polarization of Th17 cells and the activation of effector of CD8^+^ T cells. In mouse models, one species of *Bifidobacteria* could potently induce the development of intestinal Th17 cells [42,105], as shown in a study by Tan et al. that demonstrated that the human symbiont species *Bifidobacterium adolescentis* could alone induce Th17 cells in the murine intestine. Meanwhile, another study showed that *B. adolescentis* exacerbated autoimmune arthritis in a mouse model [105,106]. It was found by transcriptome analysis that *Bifidobacterium adolescentis* and SFB promoted Th17 cells in different ways, but the exact mechanisms were unclear [105,106,107]. When mice were fed with *B. longum*, significantly increased CD4**^+^** and decreased CD4^+^CD8^+^ T cell levels in the mesenteric lymph nodes and Peyer’s patches were observed [108]. Other studies showed that *Bifidobacteria* also exhibited tumor inhibition capacity whereby *Bifidobacterium*-treated mice displayed significantly improved tumor inhibition relative to non-Bifidobacterium treated counterparts. Specifically, after robust induction of tumor-specific T cells in the periphery, it was observed that the accumulation of antigen-specific CD8^+^ T cells were increased within the tumors [95].

## 4. Specific Gut Microbiota Metabolites Shapes the Balance of Th17 and Treg Cells

**Figure 2:**
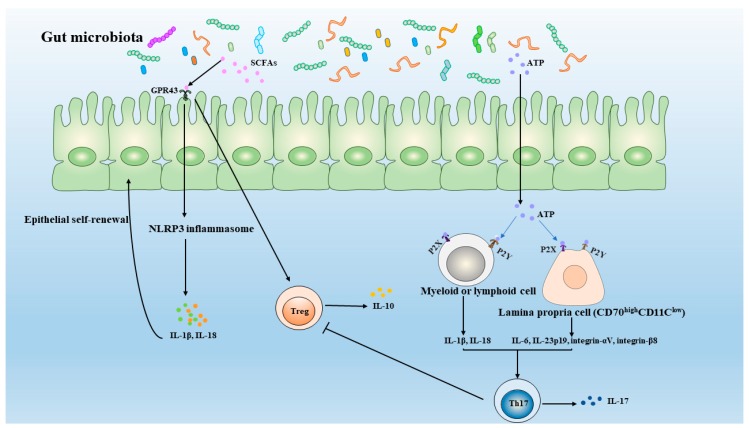
Metabolites of gut microbiota affect the balance of Th17 and Treg.

Although few microbe-host interactions have been defined at the molecular level, evidence linking metabolites of gut microbiota with the balance of Th17 and Treg cells have been reported. In these studies, adenosine ATP and SCFAs have been extensively studied, as seen in Figure 2.

SCFAs are synthesized by fermentation and decomposition of dietary fiber by anaerobic gut microbiota, which bind GPR43 to regulate the development of intestinal Treg pool and lead to NLRP3 inflammasome activation to accelerate cell maturation and secretion of IL-1β and IL-18. However, adenosine triphosphate (ATP) derived from gut microbiota via ATP sensors P2X receptors to activate Myeloid or lymphoid cell and P2Y receptors to activate Lamina propria cell (CD70^high^CD11c^low^) promote the expression of proinflammatory cytokine that promotes the development of Th17 cells and inhibit the development of Treg pool. 

### 4.1. ATP

ATP has recently been shown to modulate the functions of immune cells through ATP sensors including P2X and P2Y receptors [109]. For instance, ATP-induced P2X7R activation is critical for inflammasome-dependent IL-1β secretion [110] and high ATP concentration levels have been detected in the supernatants of in vitro-cultured intestinal commensal bacteria of SPF mice [111,112]. Meanwhile, germ-free mice possess fewer LP Th17 cells than do SPF mice. To explain the relationship between gut microbiota-derived ATP and Th17 cell differentiation and function, Atarashi et al. observed Th17 differentiation in naive CD4^+^ T cells co-cultured with lamina propria DCs in the presence of ATPγS in vitro and demonstrated that ATP derived from gut microbiota can activate a unique subset of CD70^high^CD11c^low^ lamina propria cells via P2X and P2Y receptors [111]. These cells express several molecules that lead to Th17 cell differentiation, such as IL-6, IL-23p19 and TGFβ-activating integrin-αV and -β8 [111]. Other studies have also shown that Th17 cell function is closely linked to ATP. Pandolfi et al., employing an obesity mouse model, demonstrated that ATP promotes a Th17-polarizing microenvironment with high levels of IL-1β, IL-6 and IL-17 via the P2X7 receptor pathway in visceral adipose tissue explants from lean donors. Blockade of the P2X7 receptor abrogated the levels of these cytokines [113]. As ATP is presumed to be an important factor in the pathogenesis of inflammatory bowel disease [114,115], Atarashi et al. administered ATP to mice and found that it exacerbated T-cell-mediated enterocolitis [116]. While P2X and P2Y receptors are expressed on many cells including myeloid and lymphoid cells [117], Liu et al. have shown, using a microbiota antigen-specific T cell reporter mouse system, that TLR5 mediates the induction of intestinal Th17 cells by CD172α^+^ lamina propria dendritic cells [115]. Whether gut microbiota-derived ATP can stimulate Th17 cells has not yet been demonstrated, while it is also unclear whether other cells expressing ATP receptors can mediate such function to promote Th17 cell differentiation and activation. In addition, P2X7R can be activated by extracellular ATP, leading to the assembly of NLRP3 inflammasome and the release of active IL-1β and IL-18 in a caspase-1-dependent manner [118], ultimately inhibiting IL-10 production by differentiating and memory Th17 cells [119]. However, it is unclear if ATP derived from the gut microbiota has similar functions. Thus, gut microbiota is closely tied to the development and function maturation of Th17 cells, although more future studies are needed to investigate the related mechanisms in this process.

### 4.2. SCFA (Short-Chain Fatty Acids)

Short-chain fatty acids (SCFAs) are produced by fermentation and decomposition of dietary fiber by anaerobic gut microbiota [20,120]. SCFAs play a key role in maintaining the intestinal barrier integrity [121] and can provide critical energies to colon cells for their survival [20]. In addition, SCFAs can stimulate the secretion of several antimicrobial peptides [122] and reduce the production of reactive oxygen species and proinflammatory cytokine [123]. Several amino acids released by gut bacteria can also serve as precursors for the synthesis of short-chain fatty acids [124,125]. It has been reported that SCFAs bind to free fatty acid receptor 2 (FFAR2) (GPR43) to regulate the size and function of the colonic Treg pool [126] in non-hematopoietic cells [127]. Studies have also demonstrated that gut microbiota-derived SCFAs bind to GPR43 on colonic epithelial cells to stimulate K^+^ efflux and hyperpolarization [128], leading to NACHT, LRR and PYD domains-containing protein 3 (NLRP3) inflammasome activation to accelerate cell maturation and secretion of IL-1β and IL-18 [129,130]. In addition, previous research has indicated that mice deficient in NLRP3 or certain inflammasome components are highly susceptible to dextran sodium sulfate (DSS)-induced colitis [131]. Moreover, Casp1(**^−/−^**) mice exhibited defects in mucosal tissue repair and rapidly succumbed after DSS administration [132]. These findings have also been supported by several lines of evidence, which show that increased intake of SCFAs could significantly improve experimental colitis [133,134]. Tian et al. found that during *C. rodentium* infection high levels of Th1 and Th17 cells were observed upon acetate (C2) administration, while decreased anti-CD3-induced inflammation was observed in an IL-10-dependent manner, in agreement with an earlier observation that GPR43 was expressed in colonic Tregs and myeloid cells [135]. In contrast, Park and co-authors demonstrated that T cells did not express GPR43 receptor and suggested that GPR43 did not function in regulating cytokine production in T cells, a process that is instead dependent on the activity of HDACs [136]. In fact, further research found that the HDAC inhibition in T cells by SCFAs increased the acetylation of p70 S6 kinase and the phosphorylation of rS6, with subsequent regulation of the mTOR pathway required for the generation of Th17, Th1 and IL-10^+^ T cells, a process depending on the immunological milieu. Thus, it seems that the binding of the gut microbiota-derived SCFAs to FFAR2 of non-hematopoietic cells stimulates K^+^ efflux and hyperpolarization, leads to the activation of NLRP3 inflammasomes and alteration of the local cytokine microenvironment that both regulate the size and function of the colonic Treg pool. However, direct evidence regarding the regulation of NLRP3 inflammasome and Tregs by SCFAs are still lacking and warrant further investigations. 

## 5. Discussion

Th17 and Treg cells are two vital lymphocyte subsets with opposing functions [11]. Th17 cells have important implications in autoimmune diseases and protective host immunity, while Treg cells play an important role in controlling autoimmune reactivities. Accumulated evidence suggests that the imbalance of Th17 and Treg cells is associated with the pathogenesis of many diseases including autoimmune diseases [34,35], metabolic diseases, cancer [16,17], and a balance of these two cells has been implicated in disease improvement in many cases. Gut microbiota can mount important immunomodulating effects and are critical for human health [32,33]. With the deepening of scientific research, a variety of specific gut microbiota have been found to mediate the balance of Th17 and Treg cells and the mechanisms have been studied extensively [18,19]. Considering the low cost and effective outcome of FMT in many diseases, it is tempting if FMT can be applied to diseases caused by the imbalance of Th17 and Treg cells. However, we still need to be aware of the unpredictability of this therapeutic method. Perhaps a better alternative would be a single species microorganism transfer, while this needs the elucidation of the involved mechanisms. At the same time, in order to better solve the problem of gut microbiota transplant rejection, research on the “food chain” in the microbiota ecosystem should also be taken seriously.

## Figures and Tables

**Table 1:** The function of different species Bifidobacterium strains in human immune system maturation.

Species	Function
*Bifidobacterium longum*	Up-regulate expression of IL-10, TNF-α and IFN-γ Decrease CD4^+^CD8^+^T cells Induce dendritic cell (DC) maturation
*Bifidobacterium bifidum*	Up-regulate expression of IL-17 Induce DC maturation
*Bifidobacterium animalis*	Up-regulate expression of TNF-α and IFN-γ Induce DC maturation
*Bifidobacterium adolescentis*	Alone induce Th17 cells Induce DC maturation
